# Scrub Typhus Presenting As Acute Febrile Illness With Splenic Infarct: A Rare Manifestation

**DOI:** 10.7759/cureus.45622

**Published:** 2023-09-20

**Authors:** Namita Kamra, Subramani Jagadeesan, Ramandeep Singh

**Affiliations:** 1 Internal Medicine, Vardhman Mahavir Medical College and Safdarjung Hospital, New Delhi, IND

**Keywords:** splenic infarct, complication, asia, tropical infection, scrub typhus

## Abstract

Scrub typhus is a mite-borne infectious disease endemic in India, Korea, China, Japan, Taiwan, Pakistan, Malaysia, Thailand, and Australia. It has a multitude of clinical manifestations ranging from mild symptoms like headache, myalgia, anorexia, fever, and rash to severe multiorgan failure. It can also lead to several complications, including pancreatitis, hepatitis, myocardial infarction, and cerebral infarction. A few cases of splenic infarction are also reported. We report a rare case of a 40-year-old female presenting with fever and left upper quadrant abdominal pain of acute onset. She is diagnosed serologically with scrub typhus using enzyme-linked immunosorbent assay (ELISA) after ruling out other infectious causes, including other tropical diseases. Abdominal computed tomography revealed splenic infarction attributed to scrub typhus after excluding other etiologies. She improved after a course of doxycycline and was advised to follow up. Hence, a splenic infarct should be suspected in a patient with scrub typhus complaining of acute left hypochondriac pain.

## Introduction

Scrub typhus is a mite-borne, acute febrile illness caused by *Orientia tsutsugamushi*. It has a multitude of clinical manifestations, ranging from mild non-specific symptoms to severe multiorgan failure [1,2]. An eschar may be formed, with regional lymphadenopathy occurring, which may evolve into generalized lymphadenopathy within a few days. Bacteremia and fever with a headache, cough, myalgia, and gastrointestinal symptoms are seen, usually within 8-10 days following the bite. Perivasculitis of the small vessels occurs. Endothelial cells get infected, and vascular permeability gets increased. Macrophages also get infected, due to which multiple organs can get involved, leading to fatal complications [1-3].

## Case presentation

A 40-year-old female from an urban area in North India presented to the emergency department in a tertiary healthcare center, complaining of fever for 12 days, accompanied by chills and vomiting. She also reported abdominal pain in the left upper quadrant for 10 days. She had no past history of any medical or surgical illnesses and was not on any medications. She was a homemaker and reported no exposure to any animals or plants. She gave a history of cigarette smoking for four years but quit a couple of years back. There was no history of any alcohol intake or recreational drug use. On examination, she was afebrile and vitally stable without any visible eschar marks or skin rash. Systemic examination was unremarkable, except for mild tenderness in the left hypochondrium and a palpable spleen.

**Figure 1:**
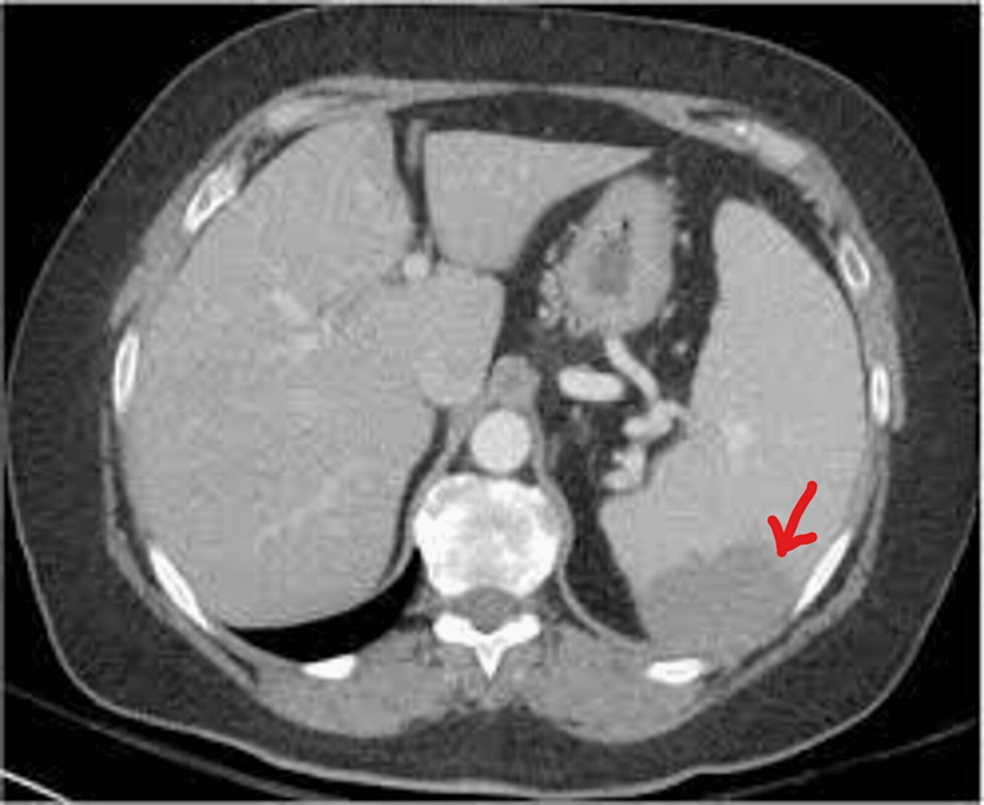
Abdominal CT showing an area of hypodense splenic infarct (as marked by red arrow)

At presentation, her hemoglobin was 11.9 gm/dl, mean corpuscular volume 89.0, total leucocyte count 12,900/cumm, and platelets 2,45,000/microliter. Her liver and kidney function tests were within normal limits. Q-CRP was positive with a value of 1.2 mg/dl. Total serum protein was 6.5 gm/dl, with serum albumin being 3.7 gm/dl. Peripheral smears of the blood were within normal limits. Dengue and malaria serologies were negative. Three blood cultures from different sites taken 24 hours apart showed no growth even after five days of incubation. Enteric fever was ruled out by serological testing and blood and stool cultures. She tested negative by enzyme-linked immunosorbent assay (ELISA) method for Leptospira antibodies. The Sickling test was negative. Hemoglobin electrophoresis was done and hemoglobinopathies were ruled out. The diagnosis of scrub typhus was confirmed using an enzyme-linked immunosorbent assay with a positive IgM antibody against *Orientia tsutsugamushi*. Hepatitis B surface antigen (HBsAg), anti-hepatitis C virus (anti-HCV), and HIV 1 and 2 antibodies were non-reactive. Infective endocarditis was ruled out by 2D echocardiography. Abdominal ultrasonography suggested mild splenic enlargement. Abdominal CT revealed mild splenomegaly with splenic infarct (Figure 1). She was managed conservatively and became afebrile within 72 hours of starting oral doxycycline. She was discharged after completing the medication course and advised to follow up.

## Discussion

Scrub typhus is a mite-borne acute febrile illness endemic in eastern and southern Asia, northern Australia, and islands of the western Pacific and Indian Oceans, which may lead to serious complications, including duodenal ulcer perforation, peritonitis, and pancreatic abscess [1,2]. Cases of acute myocardial infarction and cerebral infarction complicating scrub typhus have also been reported [3,4]. Few cases of splenic infarction associated with scrub typhus have been reported so far. Two such cases were first reported in 2004 from Korea [5]. Another case was reported in 2014 from South India, followed by another case report in 2015 from Korea [6,7]. Another few cases have been reported during 2018-2020 from India and Korea [8]. More recently in 2020, another case of a young male in India, diagnosed with splenic infarct associated with scrub typhus complicated further by a splenic abscess, was reported [9].

## Conclusions

After a thorough review of the literature, we can conclude that splenic infarction is a rare complication of scrub typhus. It remains under-reported due to a lower threshold of suspicion. Hence, a splenic infarct should be ruled out in any patient with scrub typhus complaining of acute left hypochondriac pain. Additionally, more observational studies in splenic infarct patients are warranted, especially from Western countries where scrub typhus is less prevalent, in order to determine such causal associations.
